# Long-term effects of an e-learning course on patient safety: A controlled longitudinal study with medical students

**DOI:** 10.1371/journal.pone.0210947

**Published:** 2019-01-18

**Authors:** Rainer Gaupp, Julia Dinius, Ivana Drazic, Mirjam Körner

**Affiliations:** 1 Freiburg University, Medical Faculty, Medical Psychology and Medical Sociology, Hebelstrasse, Freiburg, Germany; 2 SRH University Berlin, Ernst-Reuter-Platz, Berlin, Germany; Nord University, NORWAY

## Abstract

**Background:**

To improve patient safety, educational interventions on all system levels, including medical school are necessary. Sound theoretical knowledge on elements influencing patient safety (such as error management or team work) is the basis for behavioral changes of health care professionals.

**Methods:**

A controlled, quasi-experimental study with repeated measures was deployed. The intervention group participated in a mandatory **e**-**l**earning course on **pa**tient **s**afety (ELPAS) between October 2016 and December 2016. The control group did not receive any didactic session on patient safety. In both groups we measured technical knowledge and attitudes towards patient safety before the intervention (T0), directly after the intervention (T1) and one year after the intervention (T2). Participants were 309 third-year medical students in the intervention group and 154 first- and second-year medical students in the control group.

**Results:**

Technical knowledge in the intervention group (but not the control group) improved significantly after the intervention and remained high after one year (F(2, 84) = 13.506, p < .001, η^2^ = .243). Students of the intervention group felt better prepared for safe patient practice, even one year after the intervention F(2, 85) = 6.743, p < .002, η^2^ = .137). There was no sustainable significant effect on attitudes towards patient safety.

**Conclusion:**

This study shows, that eLearning interventions can produce significant long-term effects on patient safety knowledge, however, the study did not show long-term effects on attitudes towards patient safety. Our study implies two potential developments for future research: e-learning might be used in combination with face-to-face sessions, or more intensive (in terms of frequency and duration) e-learning sessions may be needed to achieve lasting changes in attitude.

## Introduction

Patient safety is a major concern of health-care systems worldwide.[1–6] Strategies to improve patient safety are complex and combine measures on all system levels of the health-care system,[7] e.g. infrastructure,[8] technology,[9] management,[10] processes,[11] and education[12]. Although the problem with patient safety has been known in the community for decades,[13] and countless initiatives brought remarkable success for patient safety, numerous tasks to improve patient safety remain undone, or were not successful, leaving further space for improvement.[14] One of the reasons, why it is so difficult to improve patient safety, is vulnerable, complex systems where individuals tend to blame each other and deny the existence of systemic errors.[15] The denial of systemic errors is fostered by a poor understanding of safety on one hand, [14] and by person based error management on the other hand.[16] When approaching the problem of patient safety from an educational perspective, it is therefore paramount to include sound theoretical background on error management and system theories in both undergraduate and postgraduate health care curricula, as all major patient safety curricula suggest.[17–20] Additionally to these cognitive goals, patient safety education must also focus on affective learning goals and trigger reflection on individual and team behavior to overcome blaming cultures.[21]

To address the needs for patient safety education, we developed and implemented an **e**-**l**earning course on **pa**tient **s**afety (ELPAS) at Freiburg University in 2014. This course uses a case-based interactive approach and focuses on team work,[22] error management,[23] situational awareness,[24] and crisis resource management.[25] Although e-learning courses are rarely used to approach patient safety[26], its advantages compared to traditional learning methods are promising: Constraints of time and location are irrelevant in e-learning courses, which allows higher flexibility for the learner and increases the availability of learning material.[27] Web 2.0 functionalities (including shared documents, discussion boards, wikis, chat rooms etc.) enable and foster collaborative learning and both learner-learner and learner-tutor interactions by providing technical possibilities to create content collaboratively and communicate and share content via the internet.[28, 29] Through these functionalities, the e-learning is not only a container for content, but serves as a digital hub to access and transform knowledge, discuss experiences and work on problems collaboratively with peers.

As e-learning is rarely used to teach patient safety aspects [26], we evaluate the implementation of the e-learning on patient safety (ELPAS) to determine its educational benefits and understand if e-learning can indeed be a valuable part of patient safety training. Early evaluation studies suggest that ELPAS is accepted by learners as a valuable instrument to learn about patient safety and identified variables that support the learning experience through the e-learning course.[30] A further study suggested that ELPAS has the potential to generate positive learning outcomes with regard to patient safety knowledge, metacognitive strategies and attitudes.[31] However, this earlier study was not powered to test the ELPAS group against a control group. Based on the promising results of this earlier study, we developed a more rigorous study design to test a set of hypotheses longitudinally and against a control group. Based on the theory of attitude-relevant knowledge,[32] we assume that e-learning programs can trigger changes in attitude, if the e-learning enables learners to gain specific knowledge and helps them to perceive this knowledge as attitude-relevant. Against this assumption we drafted five hypotheses. Two of them focus on attitude-relevant knowledge (i, ii), whereas the remaining three hypotheses focus on attitudes known to support patient safety (iii, iv, v):

- Learning hypothesis: Technical knowledge (specific knowledge on patient safety) increases in the intervention group after the intervention, but not in the control group.
- Preparation hypothesis: The intervention group feels better prepared for safe patient practice after the intervention, whereas no such effect is detectable in the control group.
- Monitoring hypothesis: Attitudes toward situation monitoring increase in the intervention group after the intervention, but not in the control group.
- Disclosure hypothesis: Error disclosure confidence increases in the intervention group after the intervention, but not in the control group.
- Empowerment hypothesis: Perceived importance of patient empowerment increases in the intervention group after the intervention, but not in the control group.

## Materials and methods

### Ethics statement

The study was planned and conducted in compliance with the Declaration of Helsinki on ethical principles for research involving human subjects. The protocol was approved by the local ethics committee (reg. no. 59/16). Participants were informed on the aim of the questionnaire and that data would be analyzed anonymously. Access to the questionnaires was technically impossible without opting-in to participate at the study. Written informed consent was obtained from all participants.

### Study design

A controlled, quasi-experimental study with repeated measures was deployed. The intervention group (IG) participated in a mandatory e-learning course on patient safety (ELPAS) between October 2016 and December 2016. The control group (CG) did not receive any didactic session on patient safety but followed the regular medical curriculum. As the intervention is a mandatory part of the curriculum in year 3, we allocated 3^rd^ year students to the intervention group and 1^st^ and 2^nd^ year students to the control group. In both groups we measured technical knowledge and attitudes towards patient safety before the intervention (T0), directly after the intervention (T1) and one year after the intervention (T2). We selected a 12-month interval to measure long-term effects to ensure that both, the intervention and control group, did not receive any other didactic session on patient safety which would potentially bias the study.

### Sample

**Fig 1:**
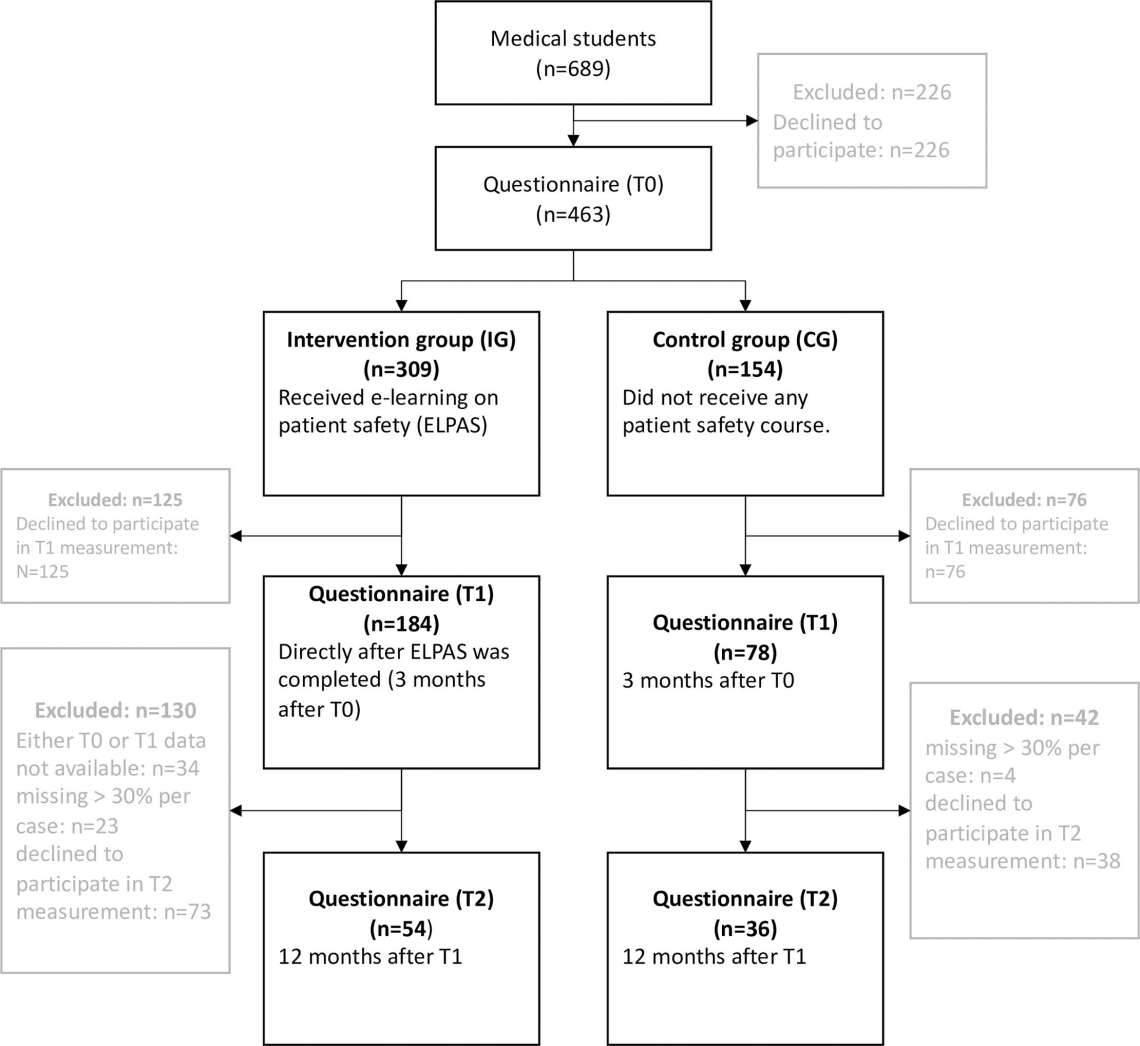
Summary of method and sample.

We pre-calculated the required sample size for the study using G*Power (vers. 3.1 for Mac OSx). Assuming small effect sizes (f = 0.2 [33]), setting α at 0.05 and targeting a statistical power of 0.8 [34], the required calculated sample size was 244. Expecting large drop outs due to the voluntary character of the study and the long-term approach, we included 689 students in the study. Of those, 309 third-year medical students in the intervention group and 154 first- and second-year medical students in the control group completed the questionnaire at T0. Complete records for T0, T1 and T2 measurements are available for 54 participants in the intervention group, and 36 participants in the control group. Fig 1 shows a flowchart of the survey population sampling.

Table 1 shows the sociodemographic data of the sample: 72.2% (n = 39) of the participants in the IG, and 69.4% (n = 25) in the CG were female (*χ*^*2*^(1, n = 64) = 0.081, p = .776). Participants of the CG were younger (72.3% (n = 26) ≤ 24 years old) than those in the IG (66.7% (n = 36) ≤ 24 years old) (*χ*^*2*^(5, n = 90) = 22.106, p < .001). 40% (n = 17) of the CG, respectively 31.5% (n = 14) of the IG had previous professional experience in health care (e.g. in nursing or midwifery) (*χ*^*2*^(4, n = 58) = 1.28, p = .864).

**Table 1:** Sociodemographic data as a percentage of the sample.

		Intervention group n = 54	Control group n = 36
		%	%
Age	17–19 years	0.0	30.6
	20–24 years	66.7	41.7
	25–29 years	20.4	19.4
	30–34 years	9.3	5.6
	35–39 years	3.7	0.0
	40–44 years	0.0	0.0
	>44 years	0.0	2.8
Gender	female	72.2	69.4
	male	27.8	30.6
Professional experience in health-care	No	68.5	60.0
	Paramedic	0.0	0.0
	Nurse	1.9	2.9
	Physiotherapist	13.0	14.3
	Midwife	13.0	14.3
	Other health profession	3.7	8.6

### Intervention

ELPAS is an online course which focuses on team work,[22] error management,[23] situational awareness,[24] and crisis resource management.[25] The content of the course is delivered in 3 major modules. German is used as the main language, although some material is delivered in English. The modules are linked to each other, but can be completed in random order. The didactic concept of the course follows a case-based interactive approach: Students work in virtual groups of 6 persons and fulfil a number of assignments including multiple choice tests, video analysis and case studies. Despite the collaborative nature of the didactic approach, students can freely choose their study time and place, as the e-learning allows both synchronous and asynchronous collaboration. [35] To complete the course, students need a total study time of 6 to 8 hours. Students have three months to complete the course. The eLearning is hosted on the online learning management system (ILIAS, Vers. 5.1, general public licence) of the university and can be accessed by any member of the university by using a course-specific password.

### Measures

Our primary outcome was technical knowledge on patient safety. Secondary outcomes were general attitudes towards patient safety and specific attitudes towards situation monitoring.

### German Version of the Attitudes Towards Patient Safety Questionnaire (GAPSQ)

The German Version of the Attitudes Towards Patient Safety Questionnaire (GAPSQ[36]) is a 14-item questionnaire to measure six dimensions of attitudes towards patient safety, using 7-point Likert scales (1 = do not agree at all; 7 = fully agree). Kiesewetter and colleagues[36] validated the questionnaire on a sample of 85 students. They found the average internal consistency reliability was Cronbachs alpha of .74. In our study, the average reliability of all GAPSQ subscales was comparable (α = .77). Means were calculated for each dimension separately; a total score is not intended for the GAPSQ. To test our hypotheses, we focused on three subscales: *Received patient safety training* measures how well students feel prepared for safe patient practice, *error reporting confidence* measures how confident students feel to report their own errors or errors they observed from other persons and *patient involvement in reducing error* measures how students rate the importance of patient empowerment for patient safety.

### TeamSTEPPS Teamwork Attitudes Questionnaire (T-TAQ)

The TeamSTEPPS Teamwork Attitudes Questionnaire (T-TAQ[37]) is a reliable and valid tool and measures individual attitudes towards major aspects of teamwork.[38] In this study we applied only the situation monitoring scale of the T-TAQ. Situation monitoring is the process of permanently monitoring behaviors and actions to assess all elements of the situation environment and its context. The scale contains six items, rated on a 5-point Likert scale (1 = do not agree; 5 = fully agree). The situation monitoring scale of the T-TAQ showed satisfactory reliability (α = .83) and discriminant validity in earlier research.[38] For our study, we translated the items to German and re-translated it for consistency checks. In our data, mean reliability (T0-T2) was acceptable (α = .69).

#### Technical knowledge

Technical knowledge was tested by a set of four single-choice questions, each presenting a short case, e.g. *“You are an emergency physician and respond to an unconscious patient*. *Two paramedics assist you*.*”*. The set of questions was developed by the authors, content validity was confirmed by an expert group. The item difficulty of the four questions ranged between .43 and .87 which is in line with the recommended item difficulty for medical examinations. [39, 40] The learner had to choose from one of five options, each suggesting a different type of more or less safe behavior. We used comparable, but not identical question sets for T0, T1, and T2. The resulting score of technical knowledge had a range of 0% (no correct answer) to 100% (4 correct answers).

### Data collection

Participants for both the intervention and control group were recruited via e-mail. Online questionnaires were distributed via the learning management system (ILIAS, Vers. 5.1, general public license). Anonymous student identity numbers were used to create paired datasets, containing T0 –T2 data. Pretest data was collected in October 2016 (T0), posttest data in January 2017 (T1) and follow-up data in January 2018 (T2).

### Data analysis

SPSS Vers. 24 (SPSS Statistics for MacOS, Version 24.0. Armonk, NY: IBM Corp.) was used for statistical analysis. Significance level was set at alpha = .05, η^2^ was calculated to report effect sizes. Chi square tests were conducted to compare sociodemographic characteristics. Analysis of covariances (ANCOVA) with repeated measurements was conducted to test the hypotheses, post-hoc simple contrasts were calculated to compare effects between the data collection points. In the analysis, we adjusted for differences in previous professional experience in healthcare, therefore we controlled for the variable professional experience.

Prior to data analysis, cases with missing values >30% were excluded.[41] For the remaining data, Little's Missing Completely At Random (MCAR) test was non-significant (*χ*^*2*^(9561) = 9667.58, *p* = .220), indicating that the data was indeed missing at random. Thus, missing values were imputed using the expectation maximization algorithm.[42]

## Results

A total of 90 participants submitted data for all three measurements from October 2016 to January 2018. Both groups (IG; CG) were comparable with regard to professional experience and gender distribution. Participants of the control group were slightly younger due to their novice status (see Table 1). In the pretest data, there were no statistically significant differences between the two groups for all outcome measures, except for the GAPSQ scale *received patient safety education* (*t*_(88)_ = 2.085, *p*<0.001, *d* = 0.46), where the control group felt slightly better prepared for safe patient actions than their peers in the intervention group (4.74; SD = .88 [CG] vs. 4.32; SD = .96 [IG]).

Results of the knowledge tests support the learning hypothesis (i): the intervention had a statistically significant effect on technical knowledge levels (F(2, 84) = 13.506, p < .001, η^2^ = .243). Technical knowledge increased significantly in the intervention group after the intervention, whereas changes in the technical knowledge of the control group remained statistically non-significant. Analysis of the follow-up measurements shows, that the knowledge differences between both groups remain significant, even after 12 months.

Along with the increase in technical knowledge, there was also a statistically significant effect of the e-learning on perceived preparedness for safe patient practice (F(2, 85) = 6.743, p < .002, η^2^ = .137), supporting the preparation hypothesis (ii). Participants of the e-learning reported an increase of perceived preparedness after the intervention and in the follow-up data, whereas the students who did not receive the e-learning felt less prepared for safe patient actions, the longer they were enrolled in the study.

**Fig 2:**
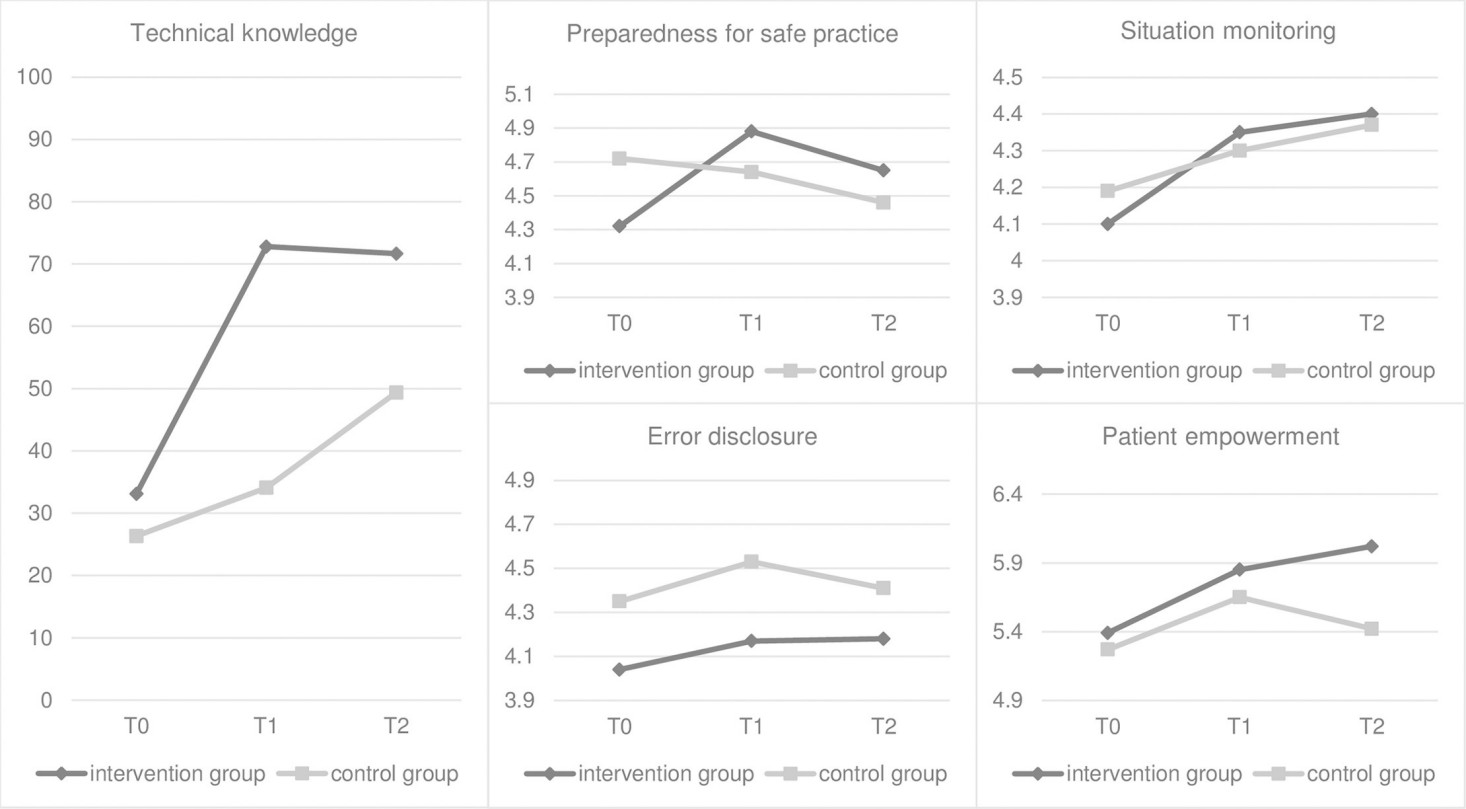
Results of the repeated measures design for T0, T1 and T2. Graphs show results of *technical knowledge*, the GAPSQ scales *preparedness for safe patient practice*, *error disclosure* and *patient empowerment* and the TTAQ scale situation monitoring.

Although there was a minor, yet statistically significant, effect on situation monitoring directly after the intervention (F(1, 198) = 9.608, p < .002, η^2^ = .046), this effect did not remain significant in the follow-up data (F(2, 85) = 1.492, p = .231), so the monitoring hypothesis (iii) is rejected. We also reject the disclosure hypothesis (iv), as the e-learning did not show any effect on error disclosing, neither in the follow-up data (T2), nor directly after the intervention(T2) (F(2, 85) = 0.160, p = .852). The data show a positive trend towards perceived higher importance of patient empowerment in the intervention group. However, this trend is statistically not significant (F(2, 81) = 2.64, p = .078) and the empowerment hypothesis is rejected (v). Fig 2 and Table 2 summarize the results:

**Table 2:** Univariate statistics for variables in analysis. IG = intervention group; CG = control group.

	Means (SE)						p
	T0		T1		T2		
	IG	CG	IG	CG	IG	CG	
Technical knowledge†	33.09 (3.10)	26.32 (3.82)	72.78 (2.89)	34.08 (3.56)	71.65 (2.86)	49.37 (3.52)	< .001
Preparedness for safe patient practice‡	4.32 (.13)	4.72 (.16)	4.88 (.13)	4.64 (.16)	4.65 (.15)	4.46 (.18)	.002
Situation Monitoring¶	4.10 (.05)	4.19 (.063)	4.35 (.06)	4.30 (.07)	4.40 (.06)	4.37 (.07)	.231
Error disclosure‡	4.04 (.16)	4.35 (.20)	4.17 (.18)	4.53 (.22)	4.18 (.16)	4.41 (.20)	.852
Patient empowerment‡	5.39 (.14)	5.27 (.17)	5.85 (.13)	5.65 (.15)	6.02 (.11)	5,42 (.14)	.078

SE: Standard error. T0: Baseline, before intervention; T1 = directly after intervention; T2 = one year after intervention.

†Technical knowledge was measured using four single choice questions. Scores represent the percentage of correct answers.

¶Situation monitoring is a subscale of the TeamSTEPPS^TM^ T-TAQ, measured on a 5-point Likert scale (5 = highest).

‡Preparedness, error disclosure and patient empowerment are three of the GAPSQ dimensions, measured on a 7-point Likert scale (7 = highest).

## Discussion

The results of this study show that ELPAS is both an effective and sustainable learning intervention. Students in the experimental group significantly improved their technical knowledge on patient safety from 33% correct answers in the pre-test to 73% in the post-test. Learning retention rates were very high, results in the follow-up test one year after the intervention were still at 72%. These results extend the existing evidence on e-learning in medical education. Although several studies proved that e-learning does help to increase knowledge, especially if enhanced interaction is embedded,[27, 43–46] very few studies have been published which demonstrate long-term effects of e-learning on knowledge particularly in the field of patient safety.[47, 48] Such long-term effects may build a stable basis for further learning. The acquired knowledge provides valuable connection points for subsequent learning contents on patient safety within the curriculum. Knowledge helps students to interpret experiences made in clinical practice and create refined knowledge by abstract conceptualization[49]. The result of the objective knowledge test is supported by subjective ratings of students on the subscales of the GAPSQ. Students participating at ELPAS feel better prepared for safe patient practice than participants in the control group. These results suggest, that students acknowledge the e-learning course and recognize that it may affect their professional practice. Patey and colleagues reported similar effects, however, they used a face-to-face didactic session on patient safety rather than an e-learning. [50]

Our data show a peak of preparedness directly after the intervention, dropping to slightly reduced values after one year. As the preparedness did not rise in the 12 months after the intervention, it is likely, that students did not receive any training in year 4 of their studies, which had direct links to patient safety.

Whereas this study supports both the learning- and the preparation hypothesis, acquisition of knowledge alone did not result in stable changes of attitudes towards patient safety. Patient empowerment[51] is not an explicit topic of the e-learning intervention, but better understanding of systems thinking and the introduction into multi-perspective analysis of errors[52] may lead to the insight, that patients are part of the system, and are, as such, relevant for patient safety. Although there is a considerable, yet statistically not significant, positive trend in the patient empowerment dimension of the GAPSQ, the empowerment hypothesis is rejected. When interpreting the data in the context of experiential learning[49], it is noticeable, that patient empowerment becomes more important in the experimental group even one year after ELPAS. Within this year students gain their first extended clinical experiences and may realize the impact patient empowerment may have on patient safety.

Contrary to the attitudes on patient empowerment, attitudes towards situation monitoring and error disclosure did not develop over time. In T1, the situation monitoring hypothesis was still supported by the data, however after one year, the effect was not evident anymore. Results of error reporting confidence led to a rejection of the disclosure hypothesis as well. Although ELPAS intensively discusses the topic, students did not change their attitude towards error disclosure. When comparing the study groups, it is obvious, that levels of error disclosure confidence are lower in the intervention group. Kiesewetter[36] showed that the levels of error disclosure confidence drops when students get more field experience in the hospital. This is also true for our intervention group; structures of hospitals may have posed a barrier for error disclosure[53].

Although we hypothesized that attitudes toward specific dimensions of patient safety may change through significant knowledge acquisition, non significant results are still not unexpected, as permanent changes in attitudes require strong stimuli.[54] Stimuli created by the e-learning may not be strong or frequent enough to permanently change attitudes, especially as the situational context (i.e. clinical experience) may not be given due to the mainly theoretical education in year 3 of the curriculum. Brown and colleagues[55] recently showed that attitudes towards quality improvement can be positively influenced by education, even in first year medical students without much clinical experience. However, their intervention comprised of facilitated workshops in small groups, rather than of e-learning. Other studies focusing on attitudes reported similar effects. Although knowledge improved, studies failed to show changes in attitudes.[56] Our study compares changes in attitudes within 15 months, i.e. a rather long time for young medical students who experience major developments in their personal and academic life within this time. Several earlier studies describe negative changes of attitudes (i.e. empathy[57], patient orientation[58] or ethical aspects[59]) in medical students, as they progress through medical school. This may impair the expected changes of patient safety related attitudes through the e-learning intervention. Although there is more recent work neglecting a negative change of attitudes during medical education[60] the fact that students gain professional experience the longer they are enrolled in medical school, often leads to less idealistic, but more realistic views on health-care.[61]

### Limitations

The outcome criteria of this study are mainly self-reported attitudes and as such subjective. Only the level of technical knowledge forms objective data. The quasi-experimental design with repeated measurements supported the interpretation of causal relationships, however, within this repeated measures design, participant drop out led to a small group remaining at T2. Based on the sample size calculation, the remaining group at T2 might not be large enough to identify small positive effects. Repeated measures with identical or similar questionnaires may also trigger learning effects, which may explain a certain knowledge acquisition even in the control group. Selection bias for both the intervention and control group are likely to have occurred, as participation was voluntary. Generalizability of the results may be limited, as the sample included only students of the medical faculty of one university. Although we used tested instruments and the reliability of the scales was acceptable, data suggest that ceiling effects cannot be ruled out.

## Conclusion

Our study shows that the e-learning achieves long-term effects on knowledge when compared to the control group. However, positive effects on attitudes, which were identified in the initial evaluation study of ELPAS, [31] could not be replicated with this more rigorous longitudinal study. In conclusion, our study implies two potential developments for future research: e-learning might be used in combination with face-to-face sessions, or more intensive (in terms of frequency and duration) e-learning sessions may be needed to achieve lasting changes in attitude.

To overcome weaknesses in the development of attitudes towards patient safety, we are currently working on an extended version of ELPAS, which shall develop patient safety competences longitudinally by short e-learning modules embedded in the medical curriculum.[62] This approach will link ELPAS much closer to medical practice and will be enhanced with simulated patient encounters and may create stimuli strong enough to lead to significant changes in attitudes.

## Supporting information

S1 File Raw data. This file contains the study raw data, except for any potentially personally identifying information.
(SAV)
